# Psychosocial determinants of physical activity among workers: an integrative review

**DOI:** 10.47626/1679-4435-2020-575

**Published:** 2021-03-03

**Authors:** Fabiane Frota da Rocha Morgado, Wesley de Souza do Vale, Claudia S. Lopes, Geraldo de Albuquerque Maranhão Neto, Eduardo Lattari, Mauro Felippe Felix Mediano, Mikael Rostila, Rosane Harter Griep, Sérgio Machado, Thaísa Alves Penna, Viviane Schultz Straatmann, Vitor Barreto Paravidino, Aldair J. Oliveira

**Affiliations:** 1 Laboratório de Dimensões Sociais Aplicadas à Atividade Física e ao Esporte, Departamento de Educação Física e Desportos, Universidade Federal Rural do Rio de Janeiro, Seropédica, RJ, Brazil; 2 Instituto de Medicina Social, Universidade do Estado do Rio de Janeiro, Rio de Janeiro, RJ, Brazil; 3 Laboratório de Ciências da Atividade Física, Universidade Salgado de Oliveira, Niterói, RJ, Brazil; 4 Kardiovize Study, International Clinical Research Centre, St. Anne’s University Hospital, Brno, Czech Republic; 5 Instituto Nacional de Infectologia Evandro Chagas, Fundação Oswaldo Cruz, Rio de Janeiro, RJ, Brazil; 6 Department of Public Health Sciences, Stockholm University, Stockholm, Sweden; 7 Laboratório de Educação em Ambiente e Saúde, Instituto Oswaldo Cruz, Fundação Oswaldo Cruz, Rio de Janeiro, RJ, Brazil; 8 Laboratório de Neurociência da Atividade Física, Programa de Pós-Graduação em Ciências da Atividade Física, Universidade Salgado de Oliveira (UNIVERSO), Niterói, RJ, Brazil; 9 Department of Neurobiology, Stockholm University, Solna, Sweden; 10 Departamento de Educação Física e Esportes, Escola Naval, Rio de Janeiro, RJ, Brazil

**Keywords:** employees, health, review, psychological factors, workplace

## Abstract

Knowledge of the psychosocial determinants of physical activity is critical to informing preventive and therapeutic interventions in the workplace. This study reviewed available evidence on psychosocial factors that have been associated with physical activity among workers. Studies were selected in December 2019 from the Scopus, Web of Science, and PubMed databases, with no date limits, using the following search terms: “physical activity”, “physical exercise”, “psychosocial”, “workers”, and “working-age”. Thirty-nine studies published between 1991 and 2019 were evaluated. The determinants of physical activity investigated among workers were smoking status, stress, psychosocial working conditions, depression, anxiety, social relationships, work ability, job satisfaction, burnout, and self-efficacy. Some consistencies and controversies were observed in the associations among these determinants and physical activity and are discussed, as are suggestions for future studies. The findings of this review may be of interest to physical activity interventions designed to reduce psychosocial risks factors in work environments.

## INTRODUCTION

Physical activity can be defined as any body movement produced by skeletal muscles that results in energy expenditure exceeding resting level^1^ and is influenced by a variety of psychological and social factors. These factors, when associated with physical practice, are considered determinants of the individual’s physical activity.^2^ Numerous studies have demonstrated that regular physical activity can improve work-related psychosocial conditions, including: stress at work,^3^ burnout, symptoms of depression and anxiety,^4^ self-efficacy,^5^ job satisfaction,^6^ and others.^7-9^ Given that physical activity increases quality of life and improves overall health,^10^ it has been widely recommended as a key component of public health policy^11^ and especially recommended for workers in a variety of workplaces.^3,12,13^

The workplace is recognized as an important context for identifying potential psychosocial risk factors for a healthy lifestyle, and several recent studies point in that direction.^^14^,15^ Sliwa et al.,^16^ for example, using a cross-sectional design, found a direct association between occupational physical activity and depressive symptoms among workers who were immigrant mothers from Latin American countries. Taking another approach, a study among workers from Switzerland by Gerber et al.^17^ showed that low levels of leisure-time physical activity (LTPA) were associated with more burnout symptoms and higher perceived stress. Nobrega et al.^18^ investigated the impact of work conditions on health in a university-community partnership by conducting eight focus groups with people holding low-wage jobs in various industries. Their results identified physical and psychosocial features of work as important antecedents of overweight. In particular, non-traditional work shifts and inflexible schedules limited participants’ ability to adhere to public health recommendations on diet and physical activity. An understanding of the particularities of different forms of work activity (e.g., shift, white-collar, blue-collar) and related psychosocial determinants, both inside and outside the workplace, may be an important step towards implementing effective preventive strategies, such as increased levels of physical activity.^19^

However, many barriers to introducing or engaging in workplace physical activity programs have been observed.^5^ The barriers hampering implementation of behavioral change have been classified by factors including individual concerns (e.g., knowledge, skills, attitudes), social context (influence of others), and environmental context (e.g., availability, climate).^20^ Scenarios characterized by adverse psychosocial factors have been observed to act as barriers to physical activity in a number of workplace contexts.^2,21,22^ Poor social relationships, for example, may be a psychosocial barrier to participating in social activities. By contrast, exercising together with colleagues may foster a positive atmosphere, which may, in turn, benefit the work environment.^5^ Thus, aspects of the psychosocial work environment should be considered in the endeavor to increase physical activity levels.

Knowledge of the psychosocial determinants associated with physical activity may contribute to increasing workers’ adherence to physical activity. This study thus reviewed available evidence on psychosocial factors that have been associated with physical activity among workers.

## METHODS

This integrative review was based on an extensive search strategy applied to the Scopus, Web of Science, and PubMed electronic databases. The search terms used were “physical activity”, “physical exercise”, “psychosocial”, “workers”, and “working-age” - all in English only. The databases were searched inclusively in “Article Title, Abstract, Keywords” (Scopus), in any “Topic” (Web of Science), or in “Any Field” (PubMed). No date limits were placed on the literature search, which was completed on December 7, 2019.

**Figure 1:**
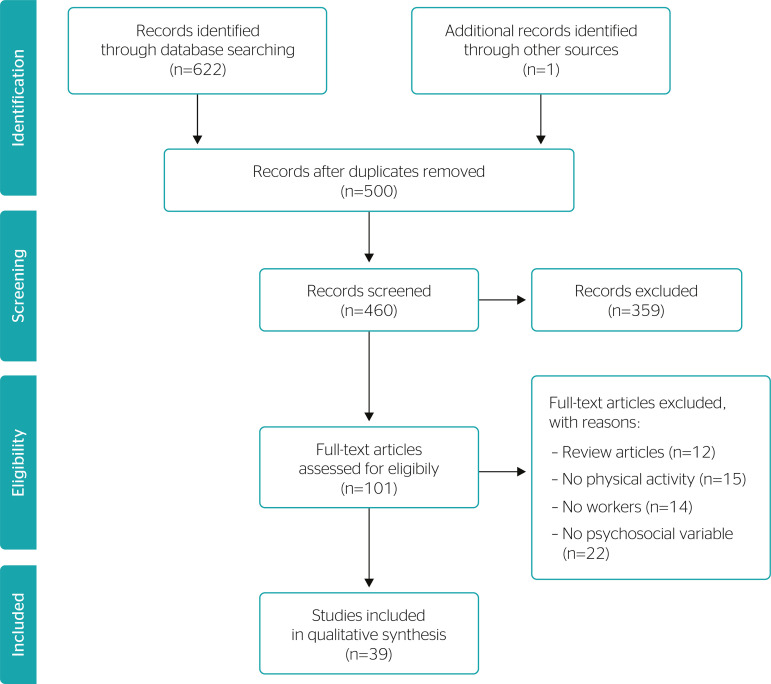
Flow diagram illustrating identification, screening, eligibility, and inclusion of studies in this review.

Articles were selected initially by examining the titles and abstracts identified during the search. Manuscripts were selected according to the inclusion criteria. Then, the full texts of the article thus selected were retrieved and evaluated against the exclusion criteria. The articles included were those that addressed psychosocial determinants associated with physical activity (e.g., total physical activity, LTPA, occupational physical activity, and physical exercise) among workers. Articles excluded were (a) those in languages other than English, Portuguese, or Spanish, (b) review articles, (c) those that drew their samples from groups other than workers or working-age persons, (d) those that did not evaluate physical activity or at least one dimension of physical activity, and e) those that did not evaluate psychosocial determinants directly. The selection process was carried out independently by a second researcher on a random sample of abstracts. Figure 1 shows a flowchart summarizing the strategy applied to identify and select studies.

Data were subjected to content analysis, strictly following three steps: a) pre-analysis (which comprised floating reading, organization, operationalization, and systematization of the material, choosing documents for analysis, and developing indicators); b) exploitation of the material: codification and thematic classification (two judges participated in this stage); and c) treatment of results, inference, and interpretation (applying descriptive analysis techniques).

## RESULTS

In all, this integrative review evaluated 39 studies. A detailed description of each publication included (author, year, country, aim, method, sample, main measures, variables, intervention, and conclusions) can be seen in Table 1.

**Table 1:** Integrative review about psychosocial determinants of physical activity in the workplace recorded in 39 included studies

	Author (country)	Aim (To investigate…)	Method	Sample	Main measures	Main variables	Intervention (period)	Main conclusions
01	Nishida et al.^2^ (Japan)	how psychological characteristics influence adoption and maintenance of PA	CsS	719 workers from manufacturing companies (MA = 43.5)	CQ (PA and others), Perceived benefit of and barriers to exercise scale	PA, self-efficacy, perceived benefit, and barriers	Not applicable	Self-efficacy, weight control benefit, physical and time barrier were psychological determinants of PA/exercise in female employees.
02	Martinez & Fischer^3^ (Brazil)	factors associated with stress at work and to verify its associations with health status among workers	CsS	474 workers from an electricity company (MA = 37.5)	CQ, The Baecke Questionnaire (PA) SF-36, Work Stress Scale, Alcohol Use Disorders Identification Test	PA, stress, mental health status	Not applicable	Special attention should be given to PA, which was inversely associated with stress at work, independently of any other factors.
03	Jonsdottir et al.^4^ (Sweden)	longitudinal associations between self-reported LTPA, perceived stress, burnout, DEP, and anxiety	LS	2,694 women; 420 men in the public service sector (MA = 47 - SD = 9.9)	CQ (PA and others), Shirom-Melamed Burnout Questionnaire, Hospital Anxiety and Depression Scale	LTPA, perceived stress, burnout, and symptoms of DEP and anxiety	Not applicable	PA reduces the risk of future mental health problems, in particular depression, burnout, and high stress levels.
04	Andersen^5^ (Denmark)	prognostic factors for adherence to workplace exercise	IS	132 office workers with neck/shoulder pain (MA = 44 (2-minute group) and 42 (12-minute group)	CQ, COPSOQ	PA, Exercise self-efficacy and psychosocial work environment	Resistance training (10 weeks)	Concurrent strategies to improve psychosocial work environment and exercise self-efficacy should be considered when implementing exercise at the workplace.
05	Andersen et al.^6^ (Denmark)	factors associated with job satisfaction in the general working population	CsS	10,427 workers (A = 18-59)	CQ	Job satisfaction, psychosocial work factors, physical demands at work, and offers of workplace health promotion	Not applicable	While psychosocial work factors and to some extent physical work demands are important for job satisfaction, workplace health-promotion offers appear to play a minor role.
06	Godin^9^ (Canada)	the psychosocial factors explaining an employee population's intention to exercise	CsS	444 workers from an electric power commission (MA = 36.3)	CQ	Intention to PA; attitude in predicting intention toward PA; perceived barriers to exercising	Not applicable	The promotion of PA at the worksite should be guided by the same principles which are applied to the promotion of habitual PA in the general population.
07	Choi et al.^11^ (United States)	associations between psychosocial work characteristics and LTPA in middle-aged US workers	CsS	2,019 workers (A = 32-69)	CQ (LTPA), JCQ, Diagnostic and Statistical Manual of Mental Disorders III-R	LTPA, DEP, SS, AI, obesity, stress, job control, and psychological job demands	Not applicable	Having on-the-job learning opportunities and decision authority on their tasks may be conducive to active LTPA in middle aged US workers.
08	Jeronimo et al.^12^ (Brazil)	temporal trends of PA among staff workers and associated factors	CsS	435 workers in psychosocial care (A = 16 ≤ 50)	Self-Report Questionnaire, IPAQ, CQ	PA, SS, BMI, DEP, anxiety	Not applicable	Interventions are needed to promote PA in this population, especially among staff workers at Centers for Psychosocial Care in smaller municipalities.
09	Kar et al.^13^ (India)	developing and implementing a healthy workplace model in a software industry of Puducherry.	IS	907 information technology workers (MA = 27.8)	CQ	PA, BMI, SS, type and nature of the job, psychosocial stress	Campaign of health promotion activities (1 year)	Dedicated and concerted efforts of the management consistent with the requirements of safety, health and environment at workplace with appropriate support from the health system can improve the quality of work and working life.
10	Marchand et al.^14^ (Canada)	diurnal sAA in association with psychosocial characteristics related to mental health, work stress, and non-work stress	CsS	395 workers from across 34 distinct workplaces (MA = 41.3 - SD = 10.81)	CQ (PA and others), BMI, General Health Questionnaire, Beck Depression Inventory, Maslach Burnout Inventory, JCQ	PA, SS, AI, BMI, mental health (psychological distress, DEP, burnout, work characteristics)	Not applicable	sAA is associated to subjective psychosocial factors, but not associated with PA.
11	Padula et al.^15^ (Brazil)	PA and work capacity in tasks with more physical exertion and others with more cognitive exertion	CsS	193 higher education workers (MA = 30) and 457 industrial workers (MA = 31)	IPAQ, WAI	PA, work ability	Not applicable	Even though workers performed tasks with different demands (cognitive versus physical), they demonstrated similar level of PA as well as work ability.
12	Sliwa et al.^16^ (United States)	relationships between occupational PA, weight-related behaviors, obesity, and DEP	CsS	385 immigrant mothers (A = 20-55)	Pregnancy Physical Activity Questionnaire, Center for Epidemiological Studies - Depression Scale	PA, DEP	Not applicable	Occupational PA contributes to energy expenditure and may protect against obesity among new immigrant mothers.
13	Gerber et al.^17^ (Switzerland)	the interaction between perceived stress, LTPA, and psychological need satisfaction on occupational burnout symptoms	CsS	306 workers (MA = 42,9 - SD = 14,1)	PSS, IPAQ-short form, Need Satisfaction Scale, Shirom-Melamed Burnout Measure	Perceived stress, LTPA, autonomy, relatedness, and competence, occupational burnout symptoms	Not applicable	Low levels of LTPA were associated with more burnout symptoms and more perceived stress.
14	Nobrega et al.^18^ (United States)	the impact of working conditions on health and weight	CsS	63 workers from various industries (A = 18-74)	Focus group	LTPA, psychosocial work stressors	Not applicable	Understanding workplace factors contributing to overweight and obesity are highly relevant to the design of effective workplace health programs, including PA ones.
15	Griep et al.^19^ (Brazil)	associations among psychosocial job strain, LTPA, and smoking in public servants	CsS	11,779 public service workers (A = 35-74)	CQ, IPAQ, Swedish Demand-Control-Support Questionnaire	PA, SS, job strain, job control, social support	Not applicable	Job strain, job control, and social support were associated with PA.
16	Oenning et al.^21^ (Brazil)	occupational factors association with major depressive disorder in workers	CsS	34,776 workers (A ≥ 18)	CQ, Patient Health Questionnaire-9	Major depressive disorder, occupational factors, practice of PA, chronic diseases, work accident	Not applicable	Intense PA at work was associated with a higher risk of major depressive disorder.
17	Andersen et al.^22^ (Denmark)	influence of physical and psychosocial working conditions on the risk of disability pension among eldercare workers	LS	4,699 female workers	CQ, COPSOQ	BMI, LTPA, psychosocial work environment and physical work environment	Not applicable	Higher level of physical exertion is a risk factor for disability pension among older female eldercare workers.
18	Dianat & Karimi^23^ (Iran)	the prevalence of musculoskeletal symptoms and associated risk factors among workers	CsS	632 workers - carpet, textiles, and leather handicraft workers (A = 18-75)	CQ, Nordic Musculoskeletal Questionnaire, observations of posture (using the Rapid Upper Limb Assessment method)	PA, SS, BMI, musculoskeletal complaints, working posture, job satisfaction	Not applicable	There is high prevalence of musculoskeletal pain among the workers. No association was found between being involved in regular sport and physical activities and the reported musculoskeletal symptoms.
19	Hanna et al.^24^ (Qatar)	relationship between levels of sedentary behavior, PA, and back pain and their psychosocial correlates among university employees	CsS	479 employees (A ≥ 25)	IPAQ, Global Physical Activity Questionnaire, Acute Low Back Pain Screening Questionnaire	PA, sedentary lifestyle and levels of back pain	Not applicable	Vigorous PA was a protective factor for back pain, therefore planned and viable strategies of PA should be incorporated into the workplace.
20	Lee et al.^25^ (United States)	interrelations among various psychosocial and behavioral variables in work	CsS	353 workers (MA = 42,8)	Burnout Scale, Depression Scale, Stress in General Scale, WAI	Exhaustion, disengagement, DEP, stress, limitations to regular physical leisure exercise, work-family balance, workability, healthy diet	Not applicable	Depressed mood was associated with less regular PA.
21	Carthy et al.^26^ (Ireland)	association between psychosocial job characteristics and health behaviors	CsS	1,025 workers at a primary health care clinic (A 50-69)	CQ, COPSOQ, IPAQ-short form, food frequency questionnaires	PA, SS, AI, perceived job characteristics	Not applicable	Positive job characteristics were associated With PA and good diet.
22	Calatayud et al.^27^ (Denmark)	association between intensity and duration of LTPA and work ability in relation to physical demands of the job	CsS	2,952 workers wage earners with physically demanding work (MA = 42)	CQ (PA and others), COPSOQ	WPA, LTPA, AI, SS, DEP psychosocial work factors	Not applicable	The duration of high-intensity PA during leisure time is associated in a dose-response fashion with work ability, in workers with physically demanding jobs.
23	Andersen et al. ^28^ (Denmark)	the effect of workplace PA on psychosocial factors among workers with chronic musculoskeletal pain	IS	66 workers (MA = 45)	General Nordic Questionnaire for Psychological and Social Factors at Work, SF-36	Social climate, vitality and mental health	Strength training (10 weeks)	Workplace physical exercise performed together with colleagues improves social climate and vitality among workers with chronic musculoskeletal pain.
24	Hallman et al.^29^ (Denmark)	different trajectories of sick leave due to musculoskeletal pain and possible associations with personal, occupational, and lifestyle factors	LS	981 workers	CQ, Danish Work Environment Cohort Survey, COPSOQ	BMI, occupational physical and psychosocial factors, LTPA, pain related factors and lifestyle factors	Not applicable	The sub-group with increasing sick leave due to pain was associated with several modifiable physical and psychosocial factors at work and outside, including LTPA.
25	Choi et al.^30^ (United States)	whether low WPA is associated with total and central obesity in male and female US workers	CsS	2,019 workers (A = 32-69)	CQ, WPA was also estimated by self-reported sedentary work and physical effort	WPA, LTPA, BMI, DEP, SS, AI, stress, sedentary work, physical job demand, psychosocial working conditions	Not applicable	Low PA at work is a significant risk factor for total and central obesity in middle-aged US male workers.
26	Sharma et al.^31^ (United States)	behavioral and psychosocial factors associated with weight status	CsS	924 hospital workers (MA = 43.6)	CQ, IPAQ	PA, sedentary behaviors, and psychosocial factors	Not applicable	Understanding the risk profile of hospital workers is critical to developing effective interventions (PA).
27	Gold et al.^32^ (United States)	factors associated with knee pain among nursing home employees	LS	4,699 workers (MA = 41.6)	CQ, JCQ	Knee pain, chronic disease, BMI, frequency of intense aerobic exercise, psychosocial work exposures, psychological job demands, social support and occupational physical exposure	Not applicable	Nursing home workers should be assisted to lose weight to protect against knee pain.
28	Yeary et al.^33^ (United States)	the impact of working conditions on health and weight	CsS	45 drivers (MA = 48.8)	Tanita BWB-800A scale, Automated Self-Administered 24-Hour Dietary Assessment, adapted IPAQ	PA, body weight, health related perceptions and attitudes, importance and support for healthy eating, PA	Not applicable	Health-related behaviors (PA) and psychosocial characteristics could serve as a basis for worksite interventions to improve drivers' health.
29	Pattussi et al.^34^ (Brazil)	the role of workplace social capital on health-related behaviors and on mental health	CsS	553 female workers from a poultry processing plant (A = 18-50)	CQ (LTPA and others), Social Capital at Work Scale, PSS, Food Frequency Questionnaire,	PA, SS, healthy eating habit, social capital, mental disorders, stress	Not applicable	Workplace social cohesion may play an important role in the promotion of mental health and healthy behaviors (PA).
30	Tonnon et al.^35^ (Netherlands)	effect of obesity on work ability in workers with high versus low physical work load	LS	36.435 male construction workers (MA = 43.9)	CQ, WAI, BMI	BMI, work ability, physical workload, health related, PA, work-related factors	Not applicable	Interventions that promote body weight loss and increase a worker's cardiorespiratory fitness might have a positive effect on work ability.
31	Emmons et al.^36^(United States)	the relationship among smoking, fat intake, and PA among workers participating in PA at the worksite	IS	1,559 manufacturing workers (MA = 41)	Paffenbarger Activity Questionnaire and Decisional Balance Scale (PA)	PA, SS, dietary fat intake	Jump Start to Health (2.5 years)	Smokers are a particular important target for health promotion intervention, and it may be possible to make initial contact with them through other health programs (PA) at workplace.
32	Sjögren et al.^37^ (Finland)	the effects of a WPA intervention on well-being	IS	90 office workers (MA = 45.7 - SD = 8.5)	CQ and Borg RPE 6-20 (WPA, LTPA) 2001	PA, self-confidence, anxiety, mood, mental stress, well-being	Resistance training (15 weeks)	Daily light resistance training, conducted during the working day, had a positive direction on subjective physical well-being.
33	Teixeira et al.^38^ (Brazil)	associations of psychosocial risk factors at work and sociodemographic and occupational characteristics with the level of PA among motorcycle taxi drivers	CsS	750 motorcycle taxi drivers (MA = 35.3)	JCQ, IPAQ	Work characteristics, psychosocial factors at work, level of PA	Not applicable	Unfavorable working conditions have an influence on the insufficient PA among motorcycle taxi drivers.
34	Jakobsen et al.^39^ (Denmark)	effect of workplace versus home-based physical exercise on psychosocial factors among health care workers	IS	200 female health care workers (MA = 42)	CQ, SF-36, COPSOQ, Disabilities of the Arm, Shoulder and Hand, Pain Catastrophizing Scale	Vitality and mental health, psychosocial work environment, work- and leisure disability, concern about pain	Strength training (10 weeks)	Performing physical exercise together with colleagues during working hours was more effective than home-based exercise in improving vitality and control of pain among health care workers.
35	Veromma et al.^40^ (Finland)	the relationship between physical health, psychosocial risk factors and work engagement among women in municipal work	CsS	726 female employees (MA = 48)	CQ, WAI	PA, SS, BMI, DEP, worker's ability to participate in work, social isolation, anxiety, hostility	Not applicable	Physical health is positively associated with work well-being driven by the positive relationship of a healthy diet and PA with work engagement.
36	Andersen et al.^41^ (Denmark)	the effect of physical exercise on social capital at work	IS	200 female health care workers (A 18-67)	Questionnaire concerning bonding, bridging, social capital; average number of training sessions	WPA, LTPA, Workplace social capital	Strength training (10 weeks)	Group-based physical exercise at work contributed to building social capital within teams at the workplace.
37	Lindstrom et al.^42^ (Sweden)	whether there are socioeconomic differences in LTPA in a Swedish population	CsS	11,837 men and women living in Malmo born 1926-1945 (A = 45-64)	CQ (LTPA)	LTPA, social network, social support, emotional support	Not applicable	It is possible that some of the socioeconomic differences in LTPA are due to differing social capital between socioeconomic groups.
38	Van den Berg et al.^43^ (Netherlands)	associations of psychosocial factors at work, lifestyle (PA), and stressful life events on health and work ability	CsS	1,141 white-collar workers in commercial services (A = 18-63)	WAI, SF-12, stress monitor, Social Readjustment Rating Questionnaire, Stanford Wellness Inventory	PA, work ability, mental and physical health, psychosocial factors at work, stressful life events and lifestyle factors	Not applicable	Psychosocial factors at work, stressful life events, lack of vigorous PA, and obesity were associated with work ability among white-collar workers.
39	Pérez-Fuentes et al.,^44^ (Spain)	implications that self-esteem, motivations for physical exercise, and eating behavior have on general wellbeing in nursing professionals	CsS	1,094 nurses (A = 22-57; MA = 32.3)	Rosenberg Self-Esteem Scale, Goal Content for Exercise Questionnaire, Three-Factor Eating Questionnaire-R18	Exercise, self-esteem, eating behavior, image, social recognition, skill development	Not applicable	Self-esteem, physical exercise and eating style were essential aspects for the health and wellbeing of workers.

A = age; AI = alcohol intake; BMI = body mass index; COPSOQ = Copenhagen Psychosocial Questionnaire; CQ = closed questions; CsS = cross-sectional study; DEP = depression; IET = individual ergonomic training; IPAQ = International Physical Activity Questionnaire; IS = intervention studies; JCQ = Job Content Questionnaire; LS = longitudinal study; LTPA = leisure-time physical activity; M = range age; MA = mean age; PA = physical activity; PSS = Perceived Stress Scale; SAA = salivary alpha-amylase; SD = standard deviation; SF-36 = Short Form Health Survey 36; SS = smoking status; ST = sedentary time; WAI = Work Ability Index; WPA = work physical activity.

Most of the studies (84.6%) were published between 2009 and 2019. The three countries with most publications included in this review were the United States (23%), Denmark (20.6%), and Brazil (18%).

In most publications (71.8%), the study used a cross-sectional method. Many studies (25.6%) used samples drawn from industrial/manufacturing workers. Workers’ ages ranged from 16 to 75 years. Sample sizes varied from 45 to 36,435 workers or working-age adults. Considering only intervention studies (n = 6), most of them (five studies) used strength training as a form of intervention. The most frequent intervention period was 10 weeks (four studies). In addition, four studies used a randomized controlled trial.

The International Physical Activity Questionnaire (IPAQ) (14.5%) was the subjective measure of physical activity most often used. The most commonly used psychosocial measures were Closed Questions (41.9%), the Copenhagen Psychosocial Questionnaire (COPSOQ) (8.2%), and the Work Ability Index (WAI) (8.2%).

Psychosocial working conditions (11.5%) and smoking status (10.6%) were the two psychosocial factors most studied, together with physical activity. Other psychosocial factors studied included stress, depression, anxiety, social relationships, work ability, job satisfaction, burnout, and self-efficacy.

## DISCUSSION

This integrative review examined psychosocial factors that have been associated with physical activity among workers. Investigation of such factors is of particular importance in relation to physical activity interventions, because workers can be affected by a number of psychosocial problems in the work environment.^12^

Our results identified records of diverse psychosocial factors associated with different dimensions of physical activity (smoking status, stress, psychosocial working, depression, anxiety, social capital, work ability, job satisfaction, burnout, and self- efficacy). They also highlighted the countries where the studies were carried out, as well as methodological aspects (methodological design, sample characteristics, instruments, and variables) and features of the interventions (type and period).

The methodological approach most used in the investigations analyzed was cross-sectional.^14,23-26^ This methodological design has several disadvantages as compared with the longitudinal study, the most important of which is that cross-sectional studies are unable to determine cause-and-effect relationships.^45^ On the other hand, longitudinal studies make it possible to follow the trajectories of psychosocial factors and physical activity over time. Although few intervention studies were identified, the physical activity intervention strategy most used was strength training. Also, interventions were found to have beneficial effects on different psychosocial factors (e.g., social climate, social capital). These findings highlight the importance of workplace physical activity as a contributory intervention towards a better working environment. It also emerged that physical exercises (e.g., strength training) could be adapted to different settings, with no need for a specific location or equipment. On the other hand, the small number of studies precluded more in-depth conclusions as to the influence of physical exercise on psychosocial determinants, as many of the determinants have not yet been investigated by intervention studies.

**Table 2:** Synthesis of the publications included in this integrative review

Variables	n	%
Year		
2009-2019	33	84.6
1998-2008	4	10.3
< 1997	2	5.1
Country		
United States	9	23.0
Denmark	8	20.6
Brazil	7	18.0
Canada	2	5.1
Sweden	2	5.1
Finland	2	5.1
Others	9	23.1
Method		
Cross-sectional study	28	71.8
Longitudinal study	5	12.8
Intervention study	6	15.4
Sample		
Industrial/manufacturing workers	10	25.6
Health care workers	6	15.3
Public servants	2	5.2
Workers with some pain	2	5.2
Others	18	48.7
Main measures		
Closed questions	26	41.9
International Physical Activity Questionnaire	9	14.5
Copenhagen Psychosocial Questionnaire	5	8.2
Short Form Health Survey 36	3	4.8
Work Ability Index	5	8.2
Depression scales (Beck Depression Inventory, Hospital Anxiety and Depression Scale, Center for Epidemiological Studies - Depression Scale)	4	6.4
Stress scales (Perceived Stress Scale, Work Stress Scale)	4	6.4
Food Frequency Questionnaire	2	3.2
Job Content Questionnaire	4	6.4
Main variables		
Physical activity/leisure-time physical activity/work physical activity	21/10/3	18.5/8.8/2.7
Smoking status	12	10.6
Stress	10	8.8
Psychosocial working conditions (environment, stressors or factors)	13	11.5
Depression	10	8.8
Body mass index	10	8.8
Anxiety	4	3.6
Social relationships (social capital, social support)	5	4.4
Work ability	3	2.7
Job satisfaction	2	1.8
Burnout	3	2.7
Self-efficacy	2	1.8
Eating habits	2	1.8
Sedentary behavior	3	2.7

The samples recruited in the studies were extremely heterogeneous as regards: a) the workers’ occupation (e.g., industrial/manufacturing, health care, workers with pain, public servants, and others); b) the workers’ age (16-75 years); and c) the sample sizes (45 to 36,435 workers). The wide variety of age ranges found in the studies was striking. It is worth remembering that these studies were conducted in different countries, with differing labor laws, which may be a plausible explanation for the variety found. The great disparity in sample size can be explained by the two methodological designs used in the investigations analyzed, since cross-sectional studies use larger samples than longitudinal studies.

In the psychosocial instruments, “closed questions” was the measure most used to evaluate variables, such as LTPA,^27^ physical exercise self-efficacy,^5^ job satisfaction,^6^ support from colleagues,^29^ and others.^7,29,46^ Results obtained with this type of measurement, when used to measure theoretically hypothetical constructs, should be interpreted with caution, since the validity and reliability of a measurement instrument are directly related to its psychometric characteristics.^47^ “Closed questions” are usually drafted by the researcher or research team and are not subjected to any rigorous psychometric procedure. Future studies should prioritize data collection instruments with proven psychometric qualities suited to the chosen worker population, so as to ensure more robust results.

The specific physical activity measure most used in the studies included in this review was the IPAQ. Choi et al.^30^ explained that methods used to evaluate physical activity are different. However, researchers have prioritized self-reported measures, which are more practical and easily applicable in large samples. Accordingly, objective measures of physical activity using accelerometers or pedometers did not appear in our approach. Analysis of the main instruments used [Closed Questions, IPAQ, COPSOQ, Short Form Health Survey 36 (SF-36), WAI, depression scales, stress scales, Food Frequency Questionnaire (FFQ), Job Content Questionnaire (JCQ)] and variables covered (physical activity/leisure time physical activity/work physical activity, smoking status, stress, psychosocial working conditions, depression, anxiety, eating habits, work ability, job satisfaction, burnout, self-efficacy, and social relationships) revealed proportional disparities among these categories. This is because one of the studies of physical activity measured it with a closed question “- How often does your job require you to sit for long periods of time during your work-shift”?^30^ -, while another study evaluated it with IPAQ.^31^

The psychosocial determinants of physical activity in the workplace were analyzed in view of the associations found between psychosocial variables studied and physical activity.^2,32^ Cross-sectional and longitudinal studies included in this review reported associations between physical activity and psychosocial factors affecting workers, including stress, psychosocial working conditions,^33^ depression,^21^ anxiety,^4^ social capital,^34^ work ability,^35^ job satisfaction,^6^ burnout,^14^ and self-efficacy.^5^

Smoking status was often a psychosocial determinant of physical activity in the studies analyzed. Smoking status (non-smoker or former smoker/current smoker) and physical (in)activity are considered to be health-related behaviors,^34^ and also the main modifiable risk factors for chronic non-communicable diseases.^19^ Smoking particularly increases risk of central obesity^11^ and is considered a cardiovascular risk factor among working populations.^13^ The close relationship between smoking and health status may be one of the main reasons for the wide interest in studying this variable among workers.

The studies included in this review pointed to a positive association between smokers and physical inactivity. Emmons,^36^ for example, highlighted the fact that, among workers, smokers were significantly more likely to engage in poor physical activity behaviors than non-smokers. Choi et al.^11^ examined whether psychosocial work characteristics and their combinations are associated with LTPA in workers. They found that non-smokers were associated strongly with active LTPA. Smokers are thus a particularly important target for health promotion interventions, which should be considered by future studies in the workplace. This suggestion is particularly important in Brazil, a country where smoking is considered to be one of the main health problems among workers.^12^

Stress was another widely studied determinant of physical activity. Psychosocial stress is considered to be a state of mental or emotional strain or tension resulting from adverse or demanding circumstances, whether in the workplace or at home. Stress has been considered a cardiovascular risk factor and is often high among workers.^13^ Workplace stressors, such as job strain, work-family interference, and fear of assault, have been linked to poor health behavior, specifically including physical inactivity.^18^

Negative associations have been observed between work stress and physical activity. Martinez and Fischer,^3^ investigating factors associated with stress at work, found that regular physical activity was inversely associated with level of stress. They explained the result by the fact that physical exercise triggers positive psychobiological changes that help to control body mass, maintain physical capacity and reduce symptoms of depression and anxiety, thus increasing self-esteem and reducing reactions to stress. Jonsdottir et al.^4^ investigated cross-sectional and longitudinal relationships between self-reported physical activity and perceived stress levels among workers. They found that individuals engaging in light physical activity and moderate-to-vigorous physical activity were less likely to report high levels of perceived stress than those reporting sedentary lifestyles. Participating in physical activity appeared to lower the risks of developing mental stress two years later. They concluded that even light physical activity could reduce stress and positively influence mental health.

On the other hand, interventions to increase physical activity proved to have less effect on stress, as found by Sjögren et al.,^37^ who examined the effects of workplace physical exercise on workers’ physical well-being, psychosocial functioning (including mental stress), and general wellbeing. They found statistically significant correspondence between strength training and increased physical well-being, but not between such training and stress. They attributed the modest results to their healthy, middle-aged volunteers’ levels of psychosocial functioning and general wellbeing already being good at baseline, and to the fact that the dose of physical exercise intervention was not high and/or prolonged enough to be effective in that sample. Future studies of workplace interventions should consider these observations.

Psychosocial working conditions (environment or stressors) are factors resulting from the individual’s interaction with the work environment, including interpersonal relations, decision authority, quantitative demands, emotional demands,^27^ job control, decision autonomy,^18^ social support from superiors, influence at work,^28^ social climate, mental health, and vitality.^6^ These psychosocial work factors may influence adherence to workplace physical activity,^5^ which may account for the broad interest of researchers in this area.

The psychosocial working environment has been shown to be an important determinant of physical activity.^28,38^ Andersen et al.^28^ found that workplace physical exercise performed together with colleagues improves the social climate and vitality among workers with chronic musculoskeletal pain. In the same direction, Jakobsen et al.^39^ found that performing physical activity together with colleagues during working hours was more effective than home-based physical exercise in improving vitality and concern about and control of pain among health care workers. However, they did not observe improvement in the sense of community among the workplace group as compared with the home group. One possible explanation they offered for this finding related to the way in which the psychosocial working environment variable was evaluated by only a single item from the COPSOQ, asking about the community at the workplace and not in the department specifically, which may have limited the quality of the results.

Depression was another recurrent psychosocial determinant in the studies examined. This construct can be conceptualized as a state of dysphoria that can vary in intensity from an oscillation in normal mood to extreme feelings of sadness, pessimism, and discouragement.^48^ There are reports in the literature that regular physical activity reduces the risk of depression,^11^ which may account for the interest in studying these variables in the working population.

Regarding the association between physical activity and depressive symptoms, the results of the studies included in this review were inconclusive. Some authors found no association between depression and physical activity among workers.^30^ Others found a negative association between these variables: Jondottir et al.,^4^ for example, found a negative association between light or moderate-to-vigorous physical activity and depressive symptoms. Other authors observed positive associations: Sliwa et al.^16^ found that occupational physical activity was associated with strong depressive symptoms, and contrasted these findings with substantial evidence from prospective studies that have shown protective effects of regular physical activity on depression. The possible explanation they offered for their finding related to other characteristics of physically demanding occupations held by immigrants, which were not measured in the study (e.g., job demands, strain, decision and scheduling control, employer discrimination). Symptoms of depression should be made the focus of future studies to build more consistent findings with regard to this especially important variable.

Anxiety is a psychosocial risk factor that associates negatively with engagement at work.^40^ It is related to a state of mood characterized by apprehension and somatic symptoms of tension, in which the individual anticipates imminent danger, catastrophe or misfortune.^48^ As regards the association of this variable with physical activity, Jondottir et al.^4^ found that participating in light or moderate-to-vigorous physical activity was significantly associated with fewer reports of anxiety symptoms, demonstrating that physical activity has a protective effect against anxiety in workers. However, the literature is contradictory on this point. Sjögren et al.,^37^ assessing a workplace physical exercise intervention, found it had no effect on anxiety. Further studies are needed to explore the association between physical activity and anxiety in the work environment and thus contribute to a more accurate understanding of these variables among workers.

Another determinant of physical activity examined was social capital, conceptualized as informal networks that facilitate cooperation within or among groups.^49^ These networks - characterized by shared norms, values, and understandings - include, for example, friends, crews, and colleagues.^41,42^ The studies included in this review agreed in highlighting the important role of social capital in keeping workers engaged in practicing physical activity. Griep et al.,^19^ for example, observed that social support at work was a protective factor for physical inactivity among women workers. Similarly, Patussi et al.^34^ found greater social capital was associated with being physically active. Researchers also emphasized the importance of physical activity to developing social capital. For example, Andersen et al.,^41^ who investigated the effect of physical exercise on social capital at work, pointed out that group-based physical exercise at work contributed to building social capital within teams at the workplace.

Work ability is determined by an individual’s perception of the demands at work and their ability to cope.^43^ It reflects the balance between individual capacity and the demands of the job. Poor work ability is associated with premature exit from the labor market, long-term sickness absence, and disability pension.^27^ The findings of this review are inconclusive regarding the association between physical activity and work ability. While some authors^27^ corroborate the hypothesis that, because physical activity increases quality of life and overall health, greater work ability would be expected among those performing high levels of physical activity, others find to the contrary.^43^ Catalayud et al.^50^ pointed out that high-intensity physical activity during leisure time was associated, in a dose-response fashion, with work ability in workers with physically demanding jobs; the duration of low intensity physical activity was not associated with work ability. In a slightly different way, Van den Berg^43^ found that the work ability of white-collar workers was strongly associated with lack of physical activity. A possible explanation for these contradictory findings may relate to the type of occupation of the workers in the above two studies.

Although job satisfaction is a very important psychosocial variable, given its consequences for the individual, workplace and society, it was little studied in the papers examined. The few findings report that physical exercise is positively, but weakly, associated with job satisfaction in the general working population.^^28^^ Future studies could focus on this variable and its association with physical activity among workers, so as to expand the limited knowledge in this area.

Burnout was another little studied variable. This phenomenon can be understood as the state of emotional exhaustion often seen as a consequence of long-term psychosocial stress.^51^ It is a dimension of mental health that it is essential to investigate in the work environment. Particularly, study of how this variable associates with physical activity could inform institutional therapy and prevention programs. However, there is a shortage of studies to evaluate this variable’s association with physical activity.^4^ Jonsdottir et al.,^4^ who evaluated the association in a sample of workers, found that participating in light and moderate-to-vigorous physical activity was significantly associated with fewer reports of burnout. They concluded that physical activity could have a preventive effect against burnout.

Self-efficacy can be conceptualized as the set of an individual’s beliefs in their ability to complete a task. It has been considered an important predictor of adherence to physical exercise in the work environment.^5^ The results from Nishida et al.^2^ indicated that self-efficacy was consistently related to physical activity. They concluded that conducting an intervention approach with female employees, especially emphasizing increased self-efficacy, was important in raising the status of physical activity. In the same way, Andersen^5^ established that self-efficacy was a prognostic factor for adherence to workplace physical activity, finding that lower adherence to a 10-week physical exercise program was predicted by lower self-efficacy. Therefore, future strategies to improve self-efficacy should be considered when implementing measures to improve physical activity levels in the workplace.

## STUDY STRENGTHS AND LIMITATIONS

This integrative review has strengths that should be highlighted. Using a search strategy and independent reviewers to identify relevant studies, it was possible to access substantial literature on the psychosocial determinants of physical activity among workers. Most of the studies included used large sample sizes and appropriate follow-up periods. The review addressed a large number of psychosocial determinants focusing on all dimensions of physical activity; to our knowledge, this has not been done previously. Nevertheless, some limitations need to be addressed. Firstly, conducting an integrative literature review meant integrating studies with major heterogeneities in design (cross-sectional, longitudinal, and intervention) and work activities. The results should thus be interpreted with caution. Furthermore, we believe that the search terms chosen were comprehensive enough to cover the large number of studies in the area with as little bias as possible. For example, choosing to prioritize only some of the possible psychosocial determinants (e.g., depression, stress, ability to work etc.) at the time of selection might have introduced bias.

## FUTURE DIRECTIONS

The findings of this integrative literature review revealed associations between psychosocial aspects and physical activity. However, most of the studies are cross-sectional. There is therefore no way to be sure of the direction of the association indicated by the studies, because of the possibility of reverse causality. Cohort studies of workers, including a range of psychosocial, health and physical activity measurements, are certainly an option in this respect, because they would be able to detect causal relationships. Furthermore, combined objective and subjective measures of physical activity should be used in future approaches.

Regarding intervention studies, future approaches could include not only proposals for physical exercise, but also strategies to improve physical activity levels throughout the work period (e.g., proposing active commuting to work) and outside the workplace, too. This may not be easy, however, as it means that working activities and the workplace must allow strategies to be implemented in this direction. In this regard, intervention studies should take into account the peculiarities of the work activities and the workplace.

## CONCLUSION

In conclusion, smoking status, stress, psychosocial working conditions, depression, anxiety, social relationships, work ability, job satisfaction, burnout, and self-efficacy are commonly studied as determinants of physical activity among workers in the workplace. Some consistencies and controversies regarding the associations between these determinants and the practice of physical activity were observed and should be carefully considered in proposed physical activity interventions designed to reduce psychosocial risk factors in work environments. Future approaches should include longitudinal designs in order to clarify the role of each psychosocial determinant in each dimension of physical activity.
